# Salivary pellets induce a pro-inflammatory response involving the TLR4–NF-kB pathway in gingival fibroblasts

**DOI:** 10.1186/s12903-016-0229-5

**Published:** 2016-07-08

**Authors:** Heinz-Dieter H-D. Müller, Barbara B. Cvikl, Adrian A. Lussi, Reinhard R. Gruber

**Affiliations:** Department of Preventive, Restorative and Pediatric Dentistry, School of Dental Medicine, University of Bern, Freiburgstrasse 7, 3010 Bern, Switzerland; Department of Conservative Dentistry and Periodontology, Medical University of Vienna, Sensengasse 2a, 1090 Vienna, Austria; Department of Oral Biology, Medical University of Vienna, Sensengasse 2a, 1090 Vienna, Austria

**Keywords:** Salivary pellet, Gingival fibroblast, Inflammation, Lipopolysaccharide, Toll-like receptor

## Abstract

**Background:**

Whole saliva provokes a substantial pro-inflammatory response in gingival fibroblasts. This raises the question whether the salivary pellet, which is used for diagnostic purposes, also has a pro-inflammatory capacity and, if yes, what the underlying mechanisms at the molecular level are.

**Methods:**

We examined the ability of extensively washed salivary pellets to provoke the expression of chemokines in gingival fibroblasts by real-time polymerase chain reaction and immunoassays. Protein composition was determined with proteomic analysis. Endotoxins were analyzed by a Limulus assay and removed by affinity chromatography. The inhibitors TAK-242 and BAY11-7082 were used to determine the involvement of the TLR4 and NF-kB signaling, respectively. Western blot was performed to detect phosphorylated p65.

**Results:**

The experiments show that salivary pellets and the corresponding washing solution contain pro-inflammatory activity without impairing cell viability. Proteomic analysis revealed proteins with a binding capacity for lipopolysaccharides, and the Limulus assay indicated the presence of endotoxin in the salivary pellets. Blocking TLR4 with TAK-242 and depletion of endotoxins both lowered the capacity of salivary pellets to increase chemokine expression and phosphorylation of p65. BAY11-7082 suppressed chemokine expression in response to the salivary pellets. Autoclaving salivary pellets also reduced their pro-inflammatory activity.

**Conclusions:**

The data support the molecular mechanism of a TLR4-NF-kB-dependent pro-inflammatory response of the gingival fibroblasts exposed to preparations of washed salivary pellets. Together, the data indicate that the salivary pellet is rich in endotoxin but it is mainly a heat labile fraction that accounts for the chemokine expression in the bioassay.

**Electronic supplementary material:**

The online version of this article (doi:10.1186/s12903-016-0229-5) contains supplementary material, which is available to authorized users.

## Background

The oral cavity is a multifunctional anatomical area representing the entrance to the gastrointestinal tract. It contributes to the gastrointestinal system through the mastication and lubrication of food in preparation for its further digestion [1]. The oral cavity houses a broad spectrum of microorganisms including both the commensal flora and pathological microorganisms that produce virulence factors [2]. Among the virulence factors are the lipopolysaccharides produced by Gram-negative bacteria, and lipoteichoic acid produced by Gram-positive bacteria [3]. Consequently, saliva not only consists of proteins produced by the salivary glands but also those produced by the virulence factors produced by bacteria [4]. Even though the presence of endotoxins, which is mainly used as a synonym for lipopolysaccharides, has long been recognized in saliva, the molecular details of the molecular mechanisms and the cellular consequences are fragmented [5, 6]. The inflammatory response induced by endotoxins has biological relevance in the development and progress of periodontal disease, where inflammation is not fully induced by changes in the subgingival environment [7, 8].

In vitro studies focusing on the cell response to sterile-filtered whole saliva revealed a robust pro-inflammatory response which resembles the response to lipopolysaccharides alone [9]. It is not clear whether lipopolysaccharides, as a fraction of whole saliva, mediate the pro-inflammatory cell response. Blocking peptides raised against the toll-like receptor (TLR) 4, which is the receptor for most endotoxins, and the downstream signaling molecule, myeloid differentiation primary response protein (MYD88), failed to reverse the pro-inflammatory activity of whole saliva [9]. Pharmacological blocking, however, revealed the involvement of nuclear factor (NF)-kB signaling [9]. Saliva aseptically obtained from the parotid gland only moderately increased the expression of pro-inflammatory cytokines [9]. Unexpectedly, it was observed that the salivary pellicle, a complex protein layer that spontaneously forms by self-assembly [10], does not provoke an early pro-inflammatory response in vitro [9]. Thus, not all salivary fractions necessarily have a pro-inflammatory potential. Currently, no information is available on the pro-inflammatory capacity of the salivary pellet, a saliva fraction that forms upon centrifugation. Therefore, this study was done to investigate the pro-inflammatory activity of the salivary pellet and to further evaluate the contribution of endotoxins as a potential stimulus for pro-inflammatory gene expression.

With advances in proteomics technology, protein data have become available on whole saliva and the salivary pellicle [11, 12], whereas the protein composition of the salivary pellet has not yet been determined. Knowing that salivary pellets can be used for diagnostic purposes, proteomics technology can help to identify potential endotoxin binding proteins. For example, pulldown experiments with proteins binding to Porphyromonas gingivalis lipopolysaccharide and Aggregatibacter actinomycetemcomitans LPS revealed numerous proteins including α-amylase, prolactin-inducible protein, and cystatin [5, 6]. This finding supports previous observations that α-amylase is one of the main components of the pro-inflammatory activity of artificial saliva [13]. The question whether the salivary pellet provokes a pro-inflammatory response in gingival fibroblasts and if this response is a result of lipopolysaccharide-binding to this protein fraction has not been investigated. In the present study we determined the pro-inflammatory response of gingival fibroblast to the salivary pellet paying particular attention to the involvement of a TLR4-mediated in vitro response.

## Methods

### Cell cultures

Primary human gingiva fibroblasts were harvested from patients who had given informed and written consent. Data will not be made available to protect the participants’ identity. The patients presented healthy gingiva without any signs of inflammation. In addition, an ethical approval was obtained from the Ethics Committee of the Medical University of Vienna (EK NR 631/2007). Gingival fibroblasts were cultured in Dulbecco’s modified Eagle medium supplemented with 10 % fetal calf serum and antibiotics (Sigma, St. Louis, USA) at 37 °C, 5 % CO_2_, and 95 % humidity. A total of three strains of fibroblasts were established and fewer than 10 passages were used for the experiments. For indicated experiments, human umbilical vein endothelial cells (HUVEC, Sigma), human oral epithelial carcinoma cells (HSC, Sigma), human osteosarcoma cells (MG63, Sigma), and human leukemic monocyte lymphoma cells (U937, Sigma) were used. For all experiments, cells were seeded at a concentration of 30,000 cells/cm^2^ onto culture dishes one day prior to stimulation.

### Collection of human salivary pellets and pellet supernatant

Human whole saliva was collected from the group of authors who were non-smokers and had no oral inflammation. They gave their written consent to publish the data. Saliva flow was stimulated by chewing paraffin wax (Ivoclar Vivadent AG, Schaan, Liechtenstein) after not eating or drinking for 1 h prior to collection. Immediately after collection, saliva was centrifuged at 4000 × g for 5 min. Saliva supernatant, which was recently tested in a bioassay [9], was discarded (Additional file 1: Figure S1). The salivary pellet was resuspended in serum-free medium (P1). For repeated washing, the salivary pellet was resuspended in serum-free medium and centrifuged up to eight times. The resuspended pellets (P1–P8) and the respective pellet supernatant (S1–S8) were collected. Preparations 1, 2, 4 and 8 were used in the bioassay (Additional file 1). For indicated experiments, LPS (100 μg/ml) and the salivary pellets (after four washes) were autoclaved at 121 °C for 20 minutes. Cells were also exposed to 100 ng/mL of recombinant proteins S100A8 and annexinA2 (ProSpec-Tany TechnoGene Ltd., Rehovot, Israel).

### Stimulation of gingival fibroblasts

Gingival fibroblasts were stimulated for 6 h with the selected preparations and subjected to RT-qPCR or viability assays. The viability measures were determined using the formazan formation assay (Sigma, St. Louis, USA), Live-Dead staining kit from Enzo Life Sciences AG (Lausen, Switzerland) and the DNA incorporation by 5-bromo-2ʹ-deoxyuridine (BrdU) Cell Proliferation ELISA kit (Roche Life Science, Penzberg, Germany).

### Reverse-transcription polymerase chain reaction and immunoassay

Total RNA was harvested with the High Pure RNA Isolation Kit (Roche, Basel, BS, Switzerland). Reverse transcription (RT) was performed with Transcriptor Universal cDNA Master (Roche). Real-time quantitative polymerase chain reaction (RT-qPCR) was done with the FastStart Universal SYBR Green Master (Roche). To quantify cDNA in the samples, the 7500 Real-Time PCR System (Applied Biosystems, Life Technologies, Carlsbad, CA) was used. Primer designing was done online via the Universal ProbeLibrary Assay Design Center (Roche) and a panel of genes for cytokines and chemokines is provided in Table 1. Relative gene expression was calculated with the RT-qPCR method [14]. The immunoassay for human CXCL8 and CXCL1 was obtained from R&D Systems (Minneapolis, MN, USA).

**Table 1:** Primer sequences of the investigated genes

Gene	Forward primer	Reverse primer	Reference
CXCL8	aacttctccacaaccctctg	ttggcagccttcctgatttc	[29]
CXCL1	tcctgcatcccccatagtta	cttcaggaacagccaccagt	[9]
CXCL2	cccatggttaagaaaatcatcg	cttcaggaacagccaccaat	[9]
IL6	gaaaggagacatgtaacaagagt	gattttcaccaggcaagtct	[30]
ICAM1	ccttcctcaccgtgtactgg	agcgtagggtaaggttcttgc	[9]

### Proteomic analysis

For mass spectrometric analysis, salivary pellet that had been washed four times (P4) was centrifuged at 16.000 × g for 1 min and the supernatant was discarded. The pellet was resuspended in 20 μl Milli-Q water (Merck Millipore, Darmstadt, Germany) and 10 μL Laemmli buffer containing 40 mM dithiothreitol, and finally boiled at 95 °C for 3 min and loaded onto a 12.5 % sodium dodecyl sulfate-polyacrylamide gel electrophoresis (SDS-PAGE). SDS-PAGE ran for 15 min, approximately 1.5 cm, and a total of seven bands were sliced and prepared to subject to the analysis. The bands were chemically reduced, alkylated and enzymatically digested with trypsin. The sample was loaded onto a pre-column (PepMap C18, 5 μm, 300 A, 300 μm × 1.5 mm length) at a flow rate of 2 μL/min with solvent A (0.1 % formic acid in water/acetonitrile 98:2). After loading, peptides were eluted in backflush mode onto the analytical Nano-column (Magic C18, 5 μm, 300 A, 0.075 mm i.d. × 75 mm length) using an acetonitrile gradient of 5 to 40 % solvent B (0.1 % formic acid in water/acetonitrile 4.9:95) for 60 min at a flow rate of 400 nL/min. The column effluent was directly coupled to an Orbitrap_XL mass spectrometer (Thermo Fischer, Bremen, Germany) via a Nanospray ESI source. Data was acquired in data dependent mode with precursor ion scans recorded in the Fourier transform detector (FT) with resolution 35.000 (at m/z = 400) parallel to five fragment spectra of the most intense precursor ions in the mass spectrometer. After the seven MS acquisition files were merged, the spectra were interpreted with Easyprot (University of Geneva, Geneva, Switzerland) on a local, dual quad core processor server run under Linux using different databases. Tolerated variable modifications were determined for carboamidomethylated Cys (no limit), Met oxidation (limited to 1), deamidation (limited to 1), phosphorylation (limited to 1). Parent and fragment mass tolerances were set to 10 ppm and 0.6 Da, respectively. All peptide identifications were filtered with two unique peptides and a false discovery rate of 1 %.

### Endotoxin detection and trapping

A Limulus amebocyte lysate assay (LAL; Life Technologies, Carlsbad, USA) was performed to quantify endotoxin levels in salivary pellets. Endotoxin removal resins were used to deplete the salivary pellets of lipopolysaccharides, according to the manufacturer’s protocol (EndoTrap HD, Hyglos, Bernried Germany). Pharmacological blocking was performed with 25 μM of TAK-242 (Merck Millipore) a TLR4 receptor inhibitor, and BAY11-7082 (Sigma), a selective and irreversible inhibitor of the TNF-α-inducible phosphorylation of IkBα.

### Western blot analysis

Human gingival fibroblasts were starved in serum-free medium overnight before being stimulated for 30 min with salivary pellet that had been washed four times. Whole human saliva, tumor necrosis factor alpha (TNF-α, 10 ng/mL) and Interleukin (IL)-1 (10 ng/mL) served as positive controls, and serum-free medium as the negative control. Cell extracts were separated by SDS-PAGE and transferred onto nitrocellulose membranes (Whatman, GE Healthcare, General Electric Company, Fairfield, CT). Primary antibody binding was accomplished with phospho-NF-kB p65 and β-actin antibodies (Cell Signaling Technology, Danvers, MA). Secondary antibody bindings were detected by near-infrared absorbing dyes with the appropriate imaging system (LI-COR Biosciences, Lincoln, NE).

### Statistical analysis

Data were compared using Friedman’s test. For post-hoc analysis the p-value was adjusted according to Dunn’s test. Statistical analysis was performed using Graph PadPrism 6.0 (GraphPad Software Inc., San Diego, USA), *p* < 0.05.

## Results

### Salivary pellets increase the expression of pro-inflammatory chemokines

**Fig. 1:**
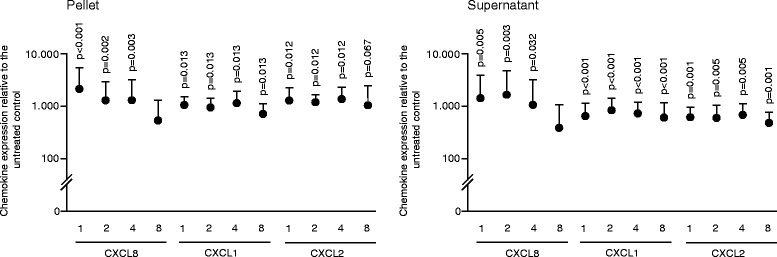
To observe chemokine expression, gingival fibroblasts were exposed to a series of dilutions of salivary pellet resuspended in serum-free medium or pellet supernatants for 6 hours. The numbers (1, 2, 4, 8) indicate the number of washing steps. A panel of chemokines was subjected to RT-qPCR assay. Data were normalized to expression levels of control cultures with serum-free medium alone. Circles represent the mean ± standard deviation of three experiments with two cell donors. Not indicated are p-values > 0.1

**Fig. 2:**
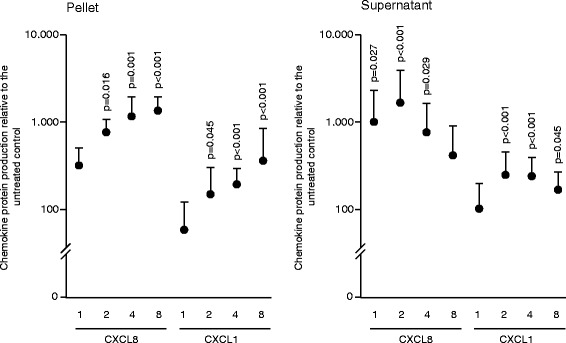
To analyze chemokine protein release, gingival fibroblasts were exposed to a series of dilutions of salivary pellet resuspended in serum-free medium or pellet supernatants for 6 hours. The numbers (1, 2, 4, 8) indicate the number of washing steps. Protein release for CXCL8 and CXCL1 was measured by immunoassays. Data were normalized to expression levels of control cultures with serum-free medium alone. Circles represent the mean ± standard deviation of three experiments with two cell donors. Not indicated are *p*-values > 0.1

**Fig. 3:**
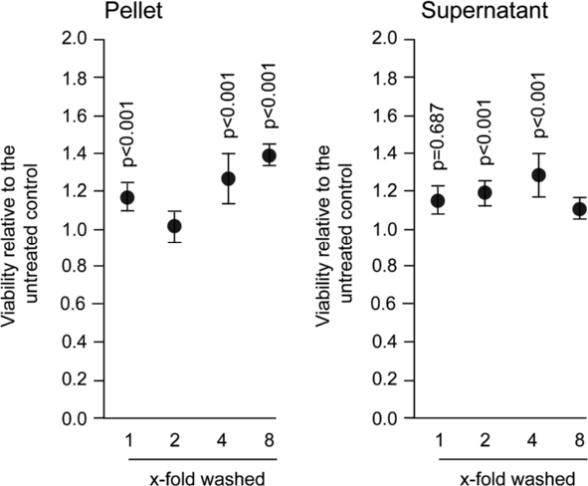
Gingival fibroblasts were exposed to different dilutions of resuspended salivary pellet or pellet supernatant for 6 hours to measure viability. Viability was determined via the conversion rate of 3-(4,5-dimethylthiazol-2-yl)-2,5-diphenyltetrazolium bromide (MTT) to formazan crystals and optical density was measured with a photometer. Data were normalized to untreated control. Circles represent the mean ± standard deviation of three experiments with two cell donors. Not indicated are *p*-values > 0.1

**Fig. 4:**
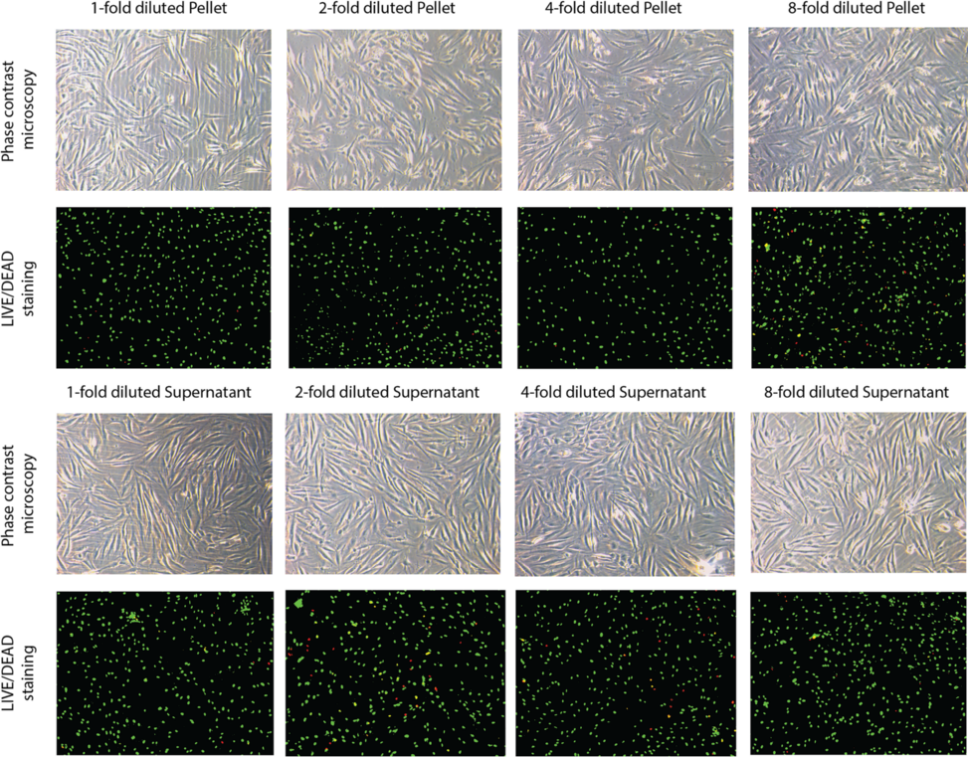
Prior phase contrast and live-dead staining microscopy gingival fibroblasts were exposed to a series of dilutions of salivary pellet and pellet supernatant, 1, 2, 4, 8 fold, respectively, for 6 h. Visual inspection using phase contrast microscopy revealed a regular fibroblastic morphology. Live-dead staining showed that most of the cells were colored green, indicating that they were vital (10 fold magnification)

**Fig. 5:**
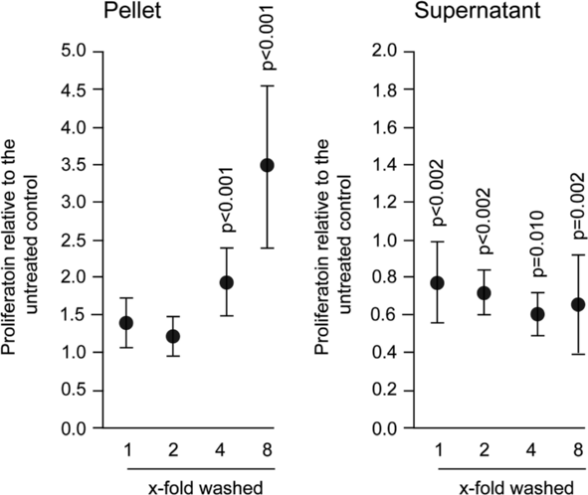
To reveal any impact on proliferation, gingival fibroblasts were exposed to a series of dilutions of salivary pellet resuspended in serum-free medium or pellet supernatants for 24 h. Proliferation was measured according to the bromdesoxyuridin (BrdU) labeling and detection protocol. Data were normalized to expression levels of control cultures with serum-free medium alone. Circles represent the mean ± standard deviation of three experiments with two cell donors. Not indicated are *p*-values > 0.1

To identify the inflammatory capacity of the salivary pellet, the expression of a panel of chemokines by gingival fibroblasts was determined. We observed a dose-dependent enhancement of CXCL8, CXCL1, and CXCL2 expression, also after repeated washings of the salivary pellet (Fig. 1). CXCL8 and CXCL1 immunoassays of the respective culture supernatant showed that the inflammatory impact was translated to the protein level (Fig. 2). CXCL8, CXCL1, and CXCL2 levels were substantially elevated in HUVEC, but not in HSC, MG63, and U937 cell lines where the expression was only marginally changed (Additional file 2). The observed pro-inflammatory response was not a result of changes in cell viability because formazan formation, live-dead staining and the incorporation of BrdU into DNA was not markedly reduced by the salivary pellets (Figs. 3, 4 and 5). Taken together, the data show that the salivary pellets have a pro-inflammatory activity and do not affect cell viability.

### Salivary pellet contains α -amylase, prolactin-inducible peptide and cystatin proteins

Based on the findings of Choi et al. and Baik et al. that salivary proteins such as α-amylase, prolactin-inducible protein, and cystatin can bind lipopolysaccharides [5, 6], proteomic analysis to identify the respective proteins in salivary pellets was performed (Additional file 3). The analysis indicated that salivary pellets contain α-amylase, prolactin-inducible protein, and cystatin, as well as other proteins with an affinity for lipopolysaccharides. Moreover, salivary pellets contain other proteins with a pro-inflammatory activity, independent of potential binding to lipopolysaccharides such as S100A8 and annexinA2 [15]. To rule out the possibility that the pro-inflammatory activity is derived from S100A8 and annexinA2, these proteins were subjected to our bioassay. As expected [16, 17], S100A8 and annexin A2 caused an approximately two-fold increase of CXCL8, CXCL1 and CXCL2 expression that, however, cannot explain the intense response of gingival fibroblasts to the salivary pellets. Proteomic analysis further revealed that salivary pellets contain considerable amounts of heat shock proteins including HSP27 and HSP70, which can provoke a pro-inflammatory response [18]. These findings support the possibility that not only do lipopolysaccharides bound to saliva proteins cause a marked pro-inflammatory, other molecules are also capable translating into inflammatory signals.

### Salivary pellets contain significant amounts of LPS

**Fig. 6:**
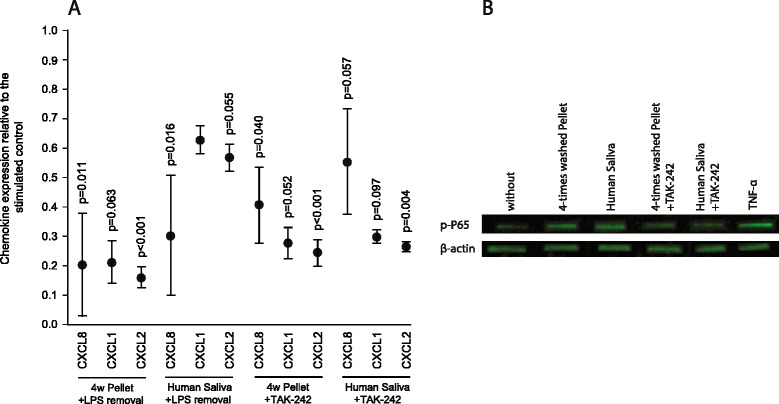
To reveal the involvement of TLR4 signaling endotoxin, removal agents were used and signal pathways were blocked. Gingival fibroblasts were exposed to salivary pellet that had been washed four times and filtered saliva for 6 hours. **a** Endotoxin removal resins reduced endotoxin-induced chemokine expression. Signal pathways were blocked with the TLR4 inhibitor TAK-242 (25 μM). **b** Salivary pellet that had been washed four times and filtered saliva increased phospho-p65 signaling in gingival fibroblasts, and TAK-242 reduced this process. Tumor necrosis factor (TNF)-α (10 ng/mL), served as positive, and serum-free medium as negative control. Data were normalized to positive expression levels of salivary pellet that had been washed four times and filtered saliva. Circles represent the mean ± standard deviation of five experiments. Not indicated are *p*-values > 0.1

To determine the amount of endotoxins in the washed salivary pellet we performed a Limulus assay. The washed salivary pellet contains more than 1900 Units/mL of endotoxin, suggesting that not only whole saliva but also the salivary pellets represent a rich source of lipopolysaccharides. To prove that the pro-inflammatory activity follows the conserved LPS–TLR4 pathway, blocking experiments were performed. Similar to earlier studies, blocking peptides against TLR4 and MYD88 failed to modulate the cell response (data not shown) [9]. However TAK-242, which is a potent pharmacological antagonist that disrupts the binding of Toll-interleukin 4 receptor (TIR) domain to its adapter molecules [19], caused a substantial suppression of CXCL8, CXCL1, and CXCL2 expression (Fig. 6a). We also used endotoxin removal resins to deplete the salivary pellets of lipopolysaccharides. The recovered salivary pellets had a significantly reduced capacity to provoke the expression of CXCL8, CXLC1, and CXLC2, compared to the original preparations (Fig. 6a). In support of previous findings with whole sterile filtered saliva [9], autoclaved salivary pellets only modestly enhanced chemokine expression of gingival fibroblasts (Table 2). Overall, the data indicate that not only the heat stable endotoxins, but also heat labile molecules are present in the washed salivary pellets, and account for the substantial chemokine expression in our bioassay.

**Table 2:** Relative chemokine expression of gingival fibroblasts in response to autoclaved salivary pellet preparations

Gene	1× washed	SD	2× washed	SD	4× washed	SD	8× washed	SD
CXCL8	9.30	8.61	15.57	9.30	28.35	22.72	25.68	12.93
CXCL1	0.83	0.44	1.24	0.52	2.20	1,28	1.74	0.67
CXCL2	1.22	0.70	1.52	0.63	2.58	1.16	2.44	1.32

Gingiva fibroblasts were stimulated with 1, 2, 4, 8 times washed autoclaved pellet preparations and chemokine expression was measured via RT-qPCR. Data represent the relative cytokine expression normalized to the untreated control. Three independent experiments with three different cell donors were performed

### Blocking TLR4 reduced chemokine expression of gingival fibroblasts

To further support the potential involvement of the TLR4 receptor pathway, the central downstream mediator, the NF-kB complex, was the focus of the next set of experiments. Western blot analysis indicated that exposure of gingival fibroblasts to washed salivary pellets causes increased phosphorylation of p65, a key component of the NF-kB complex (Fig. 6b). Blocking TLR4 with TAK-242 and depletion of lipopolysaccharides with endotoxin removal resins, both reduced the capacity of the salivary pellet to increase phosphorylation of p65 (Fig. 6a). TAK-242 also reduced chemokine expression of gingival fibroblasts exposed to sterile-filtered human saliva and salivary pellets (Table 3). Further support for the overall concept comes from studies with the pharmacological inhibitor BAY11-7082 which selectively and irreversibly blocks NF-kB activation. BAY11-7082 greatly suppressed chemokine expression in response to the salivary pellet, consistent with previous observations using whole saliva [9]. The data support the molecular mechanism of a TLR4–NF-kB-dependent pro-inflammatory response of gingival fibroblasts exposed to preparations of washed salivary pellets.

**Table 3:** Relative cytokine expression of gingival fibroblasts to sterile-filtered human saliva and salivary pellet preparations

Gene	Human saliva	SD	Human saliva + TAK-242	SD	4× washed pellet	SD	4× washed pellet + TAK-242	SD
CXCL8	2432.41*	2701.24	674.32	723.73	1438.89*	1406.80	450.75	366.06
IL6	159.02*	90.78	66.11	79.46	79.43*	50.59	36.81	27.74
ICAM1	59.90*	80.35	11.89	14.25	38.11*	46.33	8.73	8.18

Gingival fibroblasts were stimulated with 10 fold diluted *sterile-filtered* human saliva and salivary pellet preparations that had been washed four times, with or without 25 μM of TLR4-receptor inhibitor TAK-242. Data represent the relative cytokine expression normalized to the untreated control. Three independent experiments with three different cell donors were performed

* indicates *p*-values < 0.05

## Discussion

The present study aimed to help understand the in vitro response of gingival cells to saliva and its fractions. We have already reported that gingival fibroblasts respond to whole saliva with a marked pro-inflammatory response indicated by the lead chemokines CXCL8, CXCL1 and CXCL2 [9]. Among the fractions investigated was the salivary pellicle which did not induce the pro-inflammatory response after a short exposure to gingival fibroblasts in vitro [9]. The remaining questions were related to the role of the salivary pellet fraction in the pro-inflammatory response and the underlying molecular mechanisms. We showed that the salivary pellet, even after multiple washing steps, maintained its pro-inflammatory activity. By taking a series of different experimental approaches, we characterized the composition of the salivary pellet on the protein level and described how this protein fraction can serve as a carrier for endotoxins, in particular, lipopolysaccharides. Large amounts of toll-like receptor activating heat shock proteins are also found in the salivary pellet The main finding is that the salivary pellet-activated TLR4–NF-kB signaling pathway induces a substantial increase of pro-inflammatory chemokines.

Our findings, building on those of Choi et al. and Baik et al., showed that saliva proteins including α -amylase, prolactin-inducible protein and cystatin serve as carriers for endotoxins [5, 6]. These proteins were also detected in the salivary pellet by proteomic analysis; thus our data support the earlier findings. Further support for this concept comes from the finding that α-amylase was the major pro-inflammatory component of whole saliva, also depending on α-amylase enzymatic activity [13]. The present data are in line with our recent observation that the inflammatory response of gingival fibroblasts to whole saliva requires activation of NF-kB signaling, based on experiments with the inhibitor BAY11-7082 [9]. The present data do not, however, fit with the conclusions we drew based on our experiments with blocking peptides raised against the TLR4 and the downstream mediator MYD88 [9]. This discrepancy might be explained by the massive endotoxin content of saliva and of salivary pellets that likely overwhelm the inhibiting capacity of the blocking peptides. Our data do support previous observations that aseptically harvested parotid saliva showed a lower pro-inflammatory activity than whole saliva [9]. To summarize, the salivary pellet and thus also whole saliva is a rich source of endotoxins that can provoke the expected pro-inflammatory response in gingival fibroblasts.

Even though a proteomic analysis of the salivary pellet was performed, and Choi et al. and Baik et al. convincingly described a lipopolysaccharide-binding activity of proteins present in the salivary pellet, we found little evidence of binding of these composite molecules to our salivary pellets [5, 6]. Our experiments with TAK-242, however, support the involvement of TLR4, even though the inhibition is not complete. Thus, other receptors known to mediate an endotoxin response might contribute to chemokine expression in our bioassay. Moreover, it is also possible that the inhibition of TLR4 with TAK-242 is incomplete, which is supported by our findings that phosphorylation of p65 is not fully blocked by TAK-242. In addition, we have identified proteins in the salivary pellet that *per se* have a pro-inflammatory activity independent of their binding to lipopolysaccharide [20]. We selected S100A8 and annexinA2 as recombinant proteins for our tests, and observed a moderate but non-significant pro-inflammatory response. In addition large amounts of heat shock proteins were found in salivary pellets. Heat shock proteins 27 and 70 can mediate inflammation via and TLR4 [21, 22]. Yet, it is not known to what extent pro-inflammatory proteins, independent of lipopolysaccharide, contribute to the overall cellular reaction in vitro, particular considering the heat labile fraction within the salivary pellet.

In contrast to the salivary pellicle, which has a complex biological role in the oral cavity, including the remineralization of dental enamel and providing a layer for the bacterial seed that later forms plaque, the fraction of saliva that can be precipitated by centrifugation is defined by a technical process rather than a biological function. Nevertheless, fractions of salivary pellet are components of human saliva and secreted to the oral cavity via the physiological salivary flow. Clinically, the salivary pellet is used to prepare DNA for diagnostic purposes in oral cancer and arginine catabolism [23, 24]. The overall question about the possible contribution of the salivary endotoxins and those within the salivary pellet to oral homeostasis has not yet been answered. One proposal is that saliva proteins serve as a carrier of lipopolysaccharides and might support the natural process of inflammation after oral injuries. It would be interesting to find out whether sterile saliva or the respective salivary pellet can serve as a treatment to stimulate oral wound healing. This possibility is supported by observations on desalivated rats showing impaired soft and hard tissue healing in the oral cavity and also in skin wound healing [25, 26].

Future studies based on genetically modified mice models lacking TLRs would help to explain whether the pro-inflammatory activity is exclusively mediated via TLRs or other receptors mediate the cell response [27]. It might also be interesting to investigate the pathological function of LPS-binding proteins in oral diseases associated with a change in the composition of the saliva and the salivary pellet. Moreover, future studies should help us to understand the biological function of the salivary components that form the pellet. They might also investigate whether heat shock proteins and other precipitated proteins have a relevance in the development and progression of oral diseases and if they could, therefore, be useful as a diagnostic and prognostic factor in dentistry [28]. Regarding the inflammatory capacity of the salivary pellet, it might be worthwhile to investigate wound healing-related properties on local oral application. In our future studies, we aim to investigate the possible role of heat shock proteins as pro-inflammatory components in saliva.

## Conclusion

The salivary-pellet-induced pro-inflammatory response in gingival fibroblasts is mediated via the TLR4–NF-kB pathway. Chemokine expression of HUVEC was elevated on exposure to salivary pellet, while MG63, U937, and HSC cells had a differential response.

## Abbreviations

HSP, heat shock protein; IkBα, nuclear factor of ‘kappa-light-polypeptide-gene-enhancer’ in B-cells inhibitor, alpha; LPS, lipopolysaccharide; NF-kB, nuclear factor ‘kappa-light-chain-enhancer’ of activated B-cells; TLR, toll like receptor; TNF-α, tumor necrosis factor alpha

## Additional files

Additional file 1: Schematic drawing of salivary pellet processing. Gingival fibroblasts were exposed to 1, 2, 4 and 8 fold dilutions of salivary pellet resuspended in serum-free medium or supernatant obtained during pellet centrifugation for 6 h. Pellet and supernatant were harvested after centrifugation of saliva at 4000 g. After each centrifugation, the pellet was mixed by ultra-sonication. Five strains of saliva were harvested and processed (I–V). The letter P indicates the pellet harvesting and S the supernatant harvesting step. (TIF 644 kb) Additional file 2: Relative chemokine expression. Analysis of relative chemokine expression of MG63, HSC, HUVEC and U937 cells to salivary pellet and gingiva fibroblasts to lipopolysaccharides (LPS) and autoclaved LPS (aLPS). (DOCX 29 kb) Additional file 3: Proteomics analysis of salivary pellet. Systematic identification and quantification of the proteins in salivary pellets. (XLSX 6044 kb)
