# Supplementary figures and images for: Salivary pellets induce a pro-inflammatory response involving the TLR4–NF-kB pathway in gingival fibroblasts
Source: BMC Oral Health. 2016 Jul 8;17:15. doi: 10.1186/s12903-016-0229-5 (PMC4948095; doi:10.1186/s12903-016-0229-5)

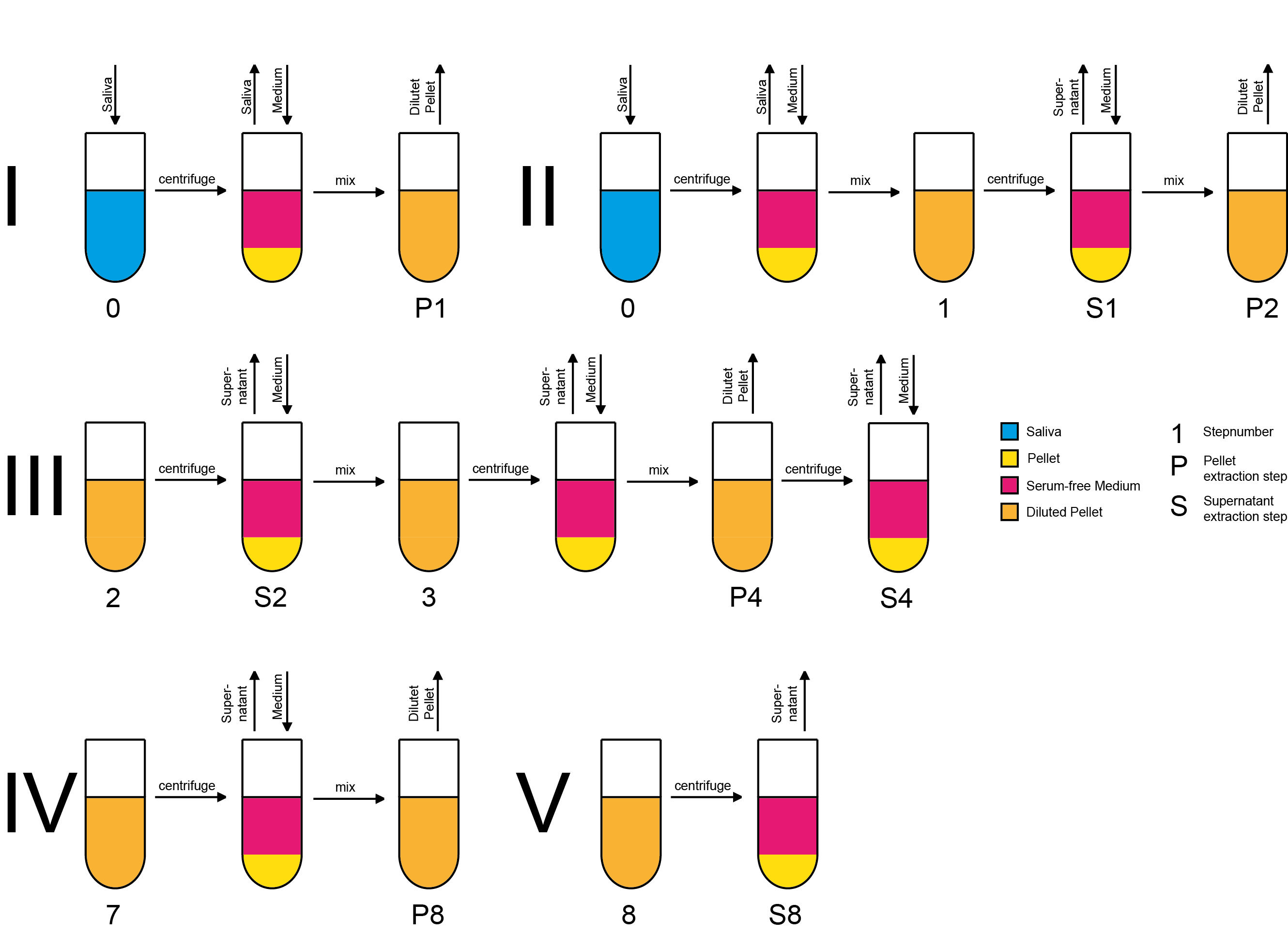

Supplement: Additional file 1: — Schematic drawing of salivary pellet processing. Gingival fibroblasts were exposed to 1, 2, 4 and 8 fold dilutions of salivary pellet resuspended in serum-free medium or supernatant obtained during pellet centrifugation for 6 h. Pellet and supernatant were harvested after centrifugation of saliva at 4000 g. After each centrifugation, the pellet was mixed by ultra-sonication. Five strains of saliva were harvested and processed (I–V). The letter P indicates the pellet harvesting and S the supernatant harvesting step. (TIF 644 kb) [file 12903_2016_229_MOESM1_ESM.tif]
