# Supplementary material for: Salivary pellets induce a pro-inflammatory response involving the TLR4–NF-kB pathway in gingival fibroblasts
Source: BMC Oral Health. 2016 Jul 8;17:15. doi: 10.1186/s12903-016-0229-5 (PMC4948095; doi:10.1186/s12903-016-0229-5)
Supplement: Additional file 2: — Relative chemokine expression. Analysis of relative chemokine expression of MG63, HSC, HUVEC and U937 cells to salivary pellet and gingiva fibroblasts to lipopolysaccharides (LPS) and autoclaved LPS (aLPS). (DOCX 29 kb) [file 12903_2016_229_MOESM2_ESM.docx]

**Additional file 2: Supplementary Tables**

*Table S1*

*Relative chemokine expression of MG63^1^, HSC^2^, HUVEC^3^ and U937^4^ to salivary pellet.*

| Pellet | 1 washed | SD | 2 washed | SD | 4 washed | SD | 8 washed | SD |
| --- | --- | --- | --- | --- | --- | --- | --- | --- |
| CXCL8^1^ | 20.51 | 15.99 | 38.08 | 41.72 | 10.98 | 16.00 | 11.05 | 13.14 |
| CXCL1^1^ | 10.77 | 13.87 | 18.88 | 24.39 | 25,92 | 30.58 | 12.73 | 12.49 |
| CXCL2^1^ | 9.58 | 8.39 | 14.46 | 14.53 | 21.35 | 18.95 | 13.58 | 7.54 |
| CXCL8^2^ | 29.27 | 25.87 | 24.92 | 21.89 | 19.65 | 16.38 | 18.28 | 15.81 |
| CXCL1^2^ | 7.50 | 8.32 | 7.75 | 6.43 | 10.54 | 8.65 | 8.79 | 7.46 |
| CXCL2^2^ | 8.05 | 5.74 | 8.56 | 4.70 | 11.01 | 5.63 | 10.19 | 2.62 |
| CXCL8^3^ | 157.82 | 135.93 | 185.82 | 117.96 | 214.80 | 285.44 | 121.64 | 172.91 |
| CXCL1^3^ | 190.93 | 152.12 | 280.20 | 243.44 | 239.86 | 141.15 | 144.48 | 127.94 |
| CXCL2^3^ | 228.54 | 108.09 | 306.39 | 199.88 | 251.87 | 202.68 | 154.67 | 154.95 |
| CXCL8^4^ | 18.28 | 3.39 | 21.90 | 3.76 | 30.03 | 10.83 | 27.67 | 2.54 |
| CXCL1^4^ | 10.57 | 6.01 | 3.92 | 0.96 | 5.68 | 2.82 | 3.63 | 3.30 |
| CXCL2^4^ | 26.32 | 17.42 | 32.19 | 23.24 | 40.00 | 30.38 | 29.77 | 18.90 |
|  |  |  |  |  |  |  |  |  |
| Supernatant | 1 washed | SD | 2 washed | SD | 4 washed | SD | 8 washed | SD |
| CXCL8^1^ | 8.18 | 7.85 | 6.37 | 9.53 | 5.74 | 7.36 | 6.11 | 4.97 |
| CXCL1^1^ | 2.99 | 2.55 | 3.29 | 3.21 | 7.51 | 5.87 | 12.94 | 17.54 |
| CXCL2^1^ | 4.72 | 3.96 | 4.04 | 4.71 | 7.72 | 6.99 | 9.37 | 10.18 |
| CXCL8^2^ | 9.40 | 7.90 | 8.50 | 8.61 | 8.48 | 7.27 | 5.76 | 4.56 |
| CXCL1^2^ | 6.62 | 6.32 | 5.18 | 4.16 | 7.29 | 5.66 | 4.16 | 2.92 |
| CXCL2^2^ | 8.89 | 9.14 | 7.26 | 5.75 | 10.17 | 8.67 | 6.29 | 5.32 |
| CXCL8^3^ | 242.68 | 147.73 | 179.35 | 157.55 | 15.11 | 5.60 | 14.37 | 7.23 |
| CXCL1^3^ | 216.09 | 92.27 | 109.60 | 40.18 | 30.65 | 7.54 | 29.19 | 20.62 |
| CXCL2^3^ | 270.57 | 113.02 | 110.76 | 29.00 | 33.05 | 9.58 | 27.37 | 12.01 |
| CXCL8^4^ | 20.28 | 9.90 | 29.87 | 15.52 | 19.65 | 6.75 | 29.78 | 21.97 |
| CXCL1^4^ | 2.30 | 1.23 | 3.57 | 1.80 | 1.19 | 0.53 | 2.96 | 1.26 |
| CXCL2^4^ | 13.94 | 9.15 | 17.83 | 9.67 | 14.07 | 7.21 | 38.63 | 29.97 |

MG63^1^, HSC^2^, HUVEC^3^ and U937^4^ were incubated with 1, 2, 4, 8 times washed whole saliva preparations and subjected to RT-qPCR to measure CXCL8, CXCL1 and CXCL2 chemokine expression. HUVEC elevated chemokine expression compared to the unstimulated control. Data represent the relative chemokine expression normalized to the untreated control. Three independent experiments with two different cell donors were performed.

*Table S2*

*Relative chemokine expression of gingiva fibroblasts to lipopolysaccharides (LPS) and autoclaved LPS (aLPS).*

| Gene | LPS 100 µg/mL | SD | aLPS 100µg/mL | SD |
| --- | --- | --- | --- | --- |
| CXCL8 | 56.71 | 42.50 | 110.56 | 40.48 |
| CXCL1 | 24.08 | 16.89 | 9.72 | 6.23 |
| CXCL2 | 19.24 | 20.49 | 12.41 | 12.86 |

Gingiva fibroblasts were stimulated with LPS (100 µg/mL) or aLPS (100 µg/mL) for 6h and chemokine expression was revealed with RT-qPCR.
